# Ensembl Genomes 2022: an expanding genome resource for non-vertebrates

**DOI:** 10.1093/nar/gkab1007

**Published:** 2021-11-13

**Authors:** Andrew D Yates, James Allen, Ridwan M Amode, Andrey G Azov, Matthieu Barba, Andrés Becerra, Jyothish Bhai, Lahcen I Campbell, Manuel Carbajo Martinez, Marc Chakiachvili, Kapeel Chougule, Mikkel Christensen, Bruno Contreras-Moreira, Alayne Cuzick, Luca Da Rin Fioretto, Paul Davis, Nishadi H De Silva, Stavros Diamantakis, Sarah Dyer, Justin Elser, Carla V Filippi, Astrid Gall, Dionysios Grigoriadis, Cristina Guijarro-Clarke, Parul Gupta, Kim E Hammond-Kosack, Kevin L Howe, Pankaj Jaiswal, Vinay Kaikala, Vivek Kumar, Sunita Kumari, Nick Langridge, Tuan Le, Manuel Luypaert, Gareth L Maslen, Thomas Maurel, Benjamin Moore, Matthieu Muffato, Aleena Mushtaq, Guy Naamati, Sushma Naithani, Andrew Olson, Anne Parker, Michael Paulini, Helder Pedro, Emily Perry, Justin Preece, Mark Quinton-Tulloch, Faye Rodgers, Marc Rosello, Magali Ruffier, James Seager, Vasily Sitnik, Michal Szpak, John Tate, Marcela K Tello-Ruiz, Stephen J Trevanion, Martin Urban, Doreen Ware, Sharon Wei, Gary Williams, Andrea Winterbottom, Magdalena Zarowiecki, Robert D Finn, Paul Flicek

**Affiliations:** European Molecular Biology Laboratory, European Bioinformatics Institute, Wellcome Genome Campus, Hinxton, Cambridge CB10 1SD, UK; European Molecular Biology Laboratory, European Bioinformatics Institute, Wellcome Genome Campus, Hinxton, Cambridge CB10 1SD, UK; European Molecular Biology Laboratory, European Bioinformatics Institute, Wellcome Genome Campus, Hinxton, Cambridge CB10 1SD, UK; European Molecular Biology Laboratory, European Bioinformatics Institute, Wellcome Genome Campus, Hinxton, Cambridge CB10 1SD, UK; European Molecular Biology Laboratory, European Bioinformatics Institute, Wellcome Genome Campus, Hinxton, Cambridge CB10 1SD, UK; European Molecular Biology Laboratory, European Bioinformatics Institute, Wellcome Genome Campus, Hinxton, Cambridge CB10 1SD, UK; European Molecular Biology Laboratory, European Bioinformatics Institute, Wellcome Genome Campus, Hinxton, Cambridge CB10 1SD, UK; European Molecular Biology Laboratory, European Bioinformatics Institute, Wellcome Genome Campus, Hinxton, Cambridge CB10 1SD, UK; European Molecular Biology Laboratory, European Bioinformatics Institute, Wellcome Genome Campus, Hinxton, Cambridge CB10 1SD, UK; European Molecular Biology Laboratory, European Bioinformatics Institute, Wellcome Genome Campus, Hinxton, Cambridge CB10 1SD, UK; Cold Spring Harbor Laboratory, 1 Bungtown Rd, Cold Spring Harbor, NY 11724, USA; European Molecular Biology Laboratory, European Bioinformatics Institute, Wellcome Genome Campus, Hinxton, Cambridge CB10 1SD, UK; European Molecular Biology Laboratory, European Bioinformatics Institute, Wellcome Genome Campus, Hinxton, Cambridge CB10 1SD, UK; Rothamsted Research, Department of Biointeractions and Crop Protection, Harpenden, Hertfordshire AL5 2JQ, UK; European Molecular Biology Laboratory, European Bioinformatics Institute, Wellcome Genome Campus, Hinxton, Cambridge CB10 1SD, UK; European Molecular Biology Laboratory, European Bioinformatics Institute, Wellcome Genome Campus, Hinxton, Cambridge CB10 1SD, UK; European Molecular Biology Laboratory, European Bioinformatics Institute, Wellcome Genome Campus, Hinxton, Cambridge CB10 1SD, UK; European Molecular Biology Laboratory, European Bioinformatics Institute, Wellcome Genome Campus, Hinxton, Cambridge CB10 1SD, UK; European Molecular Biology Laboratory, European Bioinformatics Institute, Wellcome Genome Campus, Hinxton, Cambridge CB10 1SD, UK; Department of Botany and Plant Pathology, Oregon State University, Corvallis, OR 97331, USA; European Molecular Biology Laboratory, European Bioinformatics Institute, Wellcome Genome Campus, Hinxton, Cambridge CB10 1SD, UK; Instituto de Biotecnología, Centro de Investigaciones en Ciencias Veterinarias y Agronómicas (CICVyA), Instituto Nacional de Tecnología Agropecuaria (INTA); Instituto de Agrobiotecnología y Biología Molecular (IABIMO), INTA-CONICET Nicolas Repetto y Los Reseros s/n (1686), Hurlingham, Buenos Aires, Argentina; Consejo Nacional de Investigaciones Científicas y Técnicas–CONICET, Ciudad Autónoma de Buenos Aires, Argentina; European Molecular Biology Laboratory, European Bioinformatics Institute, Wellcome Genome Campus, Hinxton, Cambridge CB10 1SD, UK; European Molecular Biology Laboratory, European Bioinformatics Institute, Wellcome Genome Campus, Hinxton, Cambridge CB10 1SD, UK; European Molecular Biology Laboratory, European Bioinformatics Institute, Wellcome Genome Campus, Hinxton, Cambridge CB10 1SD, UK; Department of Botany and Plant Pathology, Oregon State University, Corvallis, OR 97331, USA; Rothamsted Research, Department of Biointeractions and Crop Protection, Harpenden, Hertfordshire AL5 2JQ, UK; European Molecular Biology Laboratory, European Bioinformatics Institute, Wellcome Genome Campus, Hinxton, Cambridge CB10 1SD, UK; Department of Botany and Plant Pathology, Oregon State University, Corvallis, OR 97331, USA; European Molecular Biology Laboratory, European Bioinformatics Institute, Wellcome Genome Campus, Hinxton, Cambridge CB10 1SD, UK; Cold Spring Harbor Laboratory, 1 Bungtown Rd, Cold Spring Harbor, NY 11724, USA; Cold Spring Harbor Laboratory, 1 Bungtown Rd, Cold Spring Harbor, NY 11724, USA; European Molecular Biology Laboratory, European Bioinformatics Institute, Wellcome Genome Campus, Hinxton, Cambridge CB10 1SD, UK; European Molecular Biology Laboratory, European Bioinformatics Institute, Wellcome Genome Campus, Hinxton, Cambridge CB10 1SD, UK; European Molecular Biology Laboratory, European Bioinformatics Institute, Wellcome Genome Campus, Hinxton, Cambridge CB10 1SD, UK; European Molecular Biology Laboratory, European Bioinformatics Institute, Wellcome Genome Campus, Hinxton, Cambridge CB10 1SD, UK; European Molecular Biology Laboratory, European Bioinformatics Institute, Wellcome Genome Campus, Hinxton, Cambridge CB10 1SD, UK; European Molecular Biology Laboratory, European Bioinformatics Institute, Wellcome Genome Campus, Hinxton, Cambridge CB10 1SD, UK; European Molecular Biology Laboratory, European Bioinformatics Institute, Wellcome Genome Campus, Hinxton, Cambridge CB10 1SD, UK; European Molecular Biology Laboratory, European Bioinformatics Institute, Wellcome Genome Campus, Hinxton, Cambridge CB10 1SD, UK; European Molecular Biology Laboratory, European Bioinformatics Institute, Wellcome Genome Campus, Hinxton, Cambridge CB10 1SD, UK; Department of Botany and Plant Pathology, Oregon State University, Corvallis, OR 97331, USA; Cold Spring Harbor Laboratory, 1 Bungtown Rd, Cold Spring Harbor, NY 11724, USA; European Molecular Biology Laboratory, European Bioinformatics Institute, Wellcome Genome Campus, Hinxton, Cambridge CB10 1SD, UK; European Molecular Biology Laboratory, European Bioinformatics Institute, Wellcome Genome Campus, Hinxton, Cambridge CB10 1SD, UK; European Molecular Biology Laboratory, European Bioinformatics Institute, Wellcome Genome Campus, Hinxton, Cambridge CB10 1SD, UK; European Molecular Biology Laboratory, European Bioinformatics Institute, Wellcome Genome Campus, Hinxton, Cambridge CB10 1SD, UK; Department of Botany and Plant Pathology, Oregon State University, Corvallis, OR 97331, USA; European Molecular Biology Laboratory, European Bioinformatics Institute, Wellcome Genome Campus, Hinxton, Cambridge CB10 1SD, UK; Wellcome Sanger Institute, Wellcome Genome Campus, Hinxton CB10 1SA, UK; European Molecular Biology Laboratory, European Bioinformatics Institute, Wellcome Genome Campus, Hinxton, Cambridge CB10 1SD, UK; European Molecular Biology Laboratory, European Bioinformatics Institute, Wellcome Genome Campus, Hinxton, Cambridge CB10 1SD, UK; Rothamsted Research, Department of Biointeractions and Crop Protection, Harpenden, Hertfordshire AL5 2JQ, UK; European Molecular Biology Laboratory, European Bioinformatics Institute, Wellcome Genome Campus, Hinxton, Cambridge CB10 1SD, UK; European Molecular Biology Laboratory, European Bioinformatics Institute, Wellcome Genome Campus, Hinxton, Cambridge CB10 1SD, UK; European Molecular Biology Laboratory, European Bioinformatics Institute, Wellcome Genome Campus, Hinxton, Cambridge CB10 1SD, UK; Cold Spring Harbor Laboratory, 1 Bungtown Rd, Cold Spring Harbor, NY 11724, USA; European Molecular Biology Laboratory, European Bioinformatics Institute, Wellcome Genome Campus, Hinxton, Cambridge CB10 1SD, UK; Rothamsted Research, Department of Biointeractions and Crop Protection, Harpenden, Hertfordshire AL5 2JQ, UK; Cold Spring Harbor Laboratory, 1 Bungtown Rd, Cold Spring Harbor, NY 11724, USA; USDA ARS NAA Robert W. Holley Center for Agriculture and Health, Agricultural Research Service, Ithaca, NY 14853, USA; Cold Spring Harbor Laboratory, 1 Bungtown Rd, Cold Spring Harbor, NY 11724, USA; European Molecular Biology Laboratory, European Bioinformatics Institute, Wellcome Genome Campus, Hinxton, Cambridge CB10 1SD, UK; European Molecular Biology Laboratory, European Bioinformatics Institute, Wellcome Genome Campus, Hinxton, Cambridge CB10 1SD, UK; European Molecular Biology Laboratory, European Bioinformatics Institute, Wellcome Genome Campus, Hinxton, Cambridge CB10 1SD, UK; European Molecular Biology Laboratory, European Bioinformatics Institute, Wellcome Genome Campus, Hinxton, Cambridge CB10 1SD, UK; European Molecular Biology Laboratory, European Bioinformatics Institute, Wellcome Genome Campus, Hinxton, Cambridge CB10 1SD, UK

## Abstract

Ensembl Genomes (https://www.ensemblgenomes.org) provides access to non-vertebrate genomes and analysis complementing vertebrate resources developed by the Ensembl project (https://www.ensembl.org). The two resources collectively present genome annotation through a consistent set of interfaces spanning the tree of life presenting genome sequence, annotation, variation, transcriptomic data and comparative analysis. Here, we present our largest increase in plant, metazoan and fungal genomes since the project's inception creating one of the world's most comprehensive genomic resources and describe our efforts to reduce genome redundancy in our Bacteria portal. We detail our new efforts in gene annotation, our emerging support for pangenome analysis, our efforts to accelerate data dissemination through the Ensembl Rapid Release resource and our new AlphaFold visualization. Finally, we present details of our future plans including updates on our integration with Ensembl, and how we plan to improve our support for the microbial research community. Software and data are made available without restriction via our website, online tools platform and programmatic interfaces (available under an Apache 2.0 license). Data updates are synchronised with Ensembl's release cycle.

## INTRODUCTION

Ensembl Genomes (https://www.ensemblgenomes.org) provides access and analysis for non-vertebrate genomes across the domain of life. It is organised around the five kingdoms of life: plants (https://plants.ensembl.org), invertebrate metazoans (https://metazoa.ensembl.org), fungi (https://fungi.ensembl.org), protists (https://protists.ensembl.org) and bacteria (https://bacteria.ensembl.org). These five resources complement the Ensembl project (1) (https://www.ensembl.org), whose focus is vertebrate metazoans and model organisms.

As previously reported, we provide high-quality annotated genome assemblies, integrate and link with other complementary genome resources, represent genomic diversity and deliver a comprehensive analysis platform (2). We provide secondary analysis platforms including whole genome pairwise and multiple sequence alignment, homology prediction and transcriptomic analysis, ontology-based gene annotations and pathway associations. Our secondary analyses are enabled by a shared data representation and infrastructure with Ensembl, meaning tools and analysis methods developed for vertebrates are compatible with the non-vertebrate genomes with minimal, or no, modification required.

All genome assemblies are imported from the International Nucleotide Sequence Database Collaboration (INSDC) (3). Only INSDC accessioned sequences are hosted as part of our joint browser agreement with NCBI (4) and UCSC(5). We also import variation data sets from the European Variation Archive (EVA) (https://www.ebi.ac.uk/eva/) and provide automated alignment of plant transcriptome data as submitted to the European Nucleotide Archive (ENA) (6) through our collaboration with Expression Atlas (7). Our resources are further enhanced by our active collaborations with other major non-vertebrate genome providers including Gramene for plant genomes of crops, models, and species of evolutionary importance (8), VEUPathDB for eukaryotic pathogens (9) and invertebrate vectors of disease-causing pathogens (10), WormBase providing for nematodes and flatworms (11) and PHI-base for manually curated pathogen-host interactions (12).

Genomes can be accessed via one of our dedicated taxonomic websites or through the Ensembl Rapid Release resource (https://rapid.ensembl.org). All Ensembl sites provide genome browsing functionality; a way to explore the spatial relationships between annotated genomic elements. Functional annotation of genes, transcripts and proteins are enabled through imports of UniProt curated functions (13), imputation from sequence analysis tools such as InterProScan (14) and imports of manual curation of host-pathogen interactions from PHI-base. We provide comparative genomic analysis including whole genome alignments and gene orthology prediction (available for all eukaryotic taxonomic divisions), a pan-taxonomic gene orthology prediction covering key species across the tree of life and PANTHER based classification of bacterial gene families (15). Search and BLAST is available for all genomes (16). A public MySQL database server, Perl and RESTful Application Programming Interfaces (APIs) (https://rest.ensembl.org), BioMart (17) and bulk access flat-files (ftp.ensemblgenomes.org) is available for all genomes hosted in our taxonomic sites. Genomes can be analysed with standard Ensembl tools such as the Ensembl Variant Effect Predictor (VEP) (18). Each taxon-specific website is archived once per year with releases 45 (e.g. https://eg45-plants.ensembl.org/) and 49 (e.g. https://eg49-plants.ensembl.org/) being nominated for archive in 2019 and 2020, respectively. Genomes provided via Rapid Release, described later, only have a genome browser, minimal functional data imports, BLAST and flat-file access via Ensembl's FTP site (ftp.ensembl.org/pub/rapid-release/species/). All data generated by Ensembl Genomes are available for use without restriction.

Since our last review, we have seen one of the largest increases in eukaryotic genomes available through our platform with over 500 new species. As the number of genomes increased, we have had to adapt both our infrastructure and analyses to ensure scalability, continue to provide world-class genomic annotations and make available new data visualizations. Below, we highlight the new genomes and features that have been introduced over the last two years.

## NEW AND IMPROVED GENOMES

The past two years have seen significant increases in our plant, metazoan and fungal genome collections (see Table 1), totalling 588 additional genomes. We have expanded our taxonomic breadth of plants, which now includes asterids (e.g. sesame, lettuce), grasses (barley and wheat cultivars) and *Brassicaceae* (false flax and alpine rock-cress). Thirteen tree genomes have been added including *Pistacia vera* (pistachio), *Olea europaea* (olive tree), *Corylus avellana* (common hazel), *Eucalyptus grandis* (eucalyptus) and *Quercus lobata* (Valley Oak). Many of these species have a long generation time, as in the case of *Corylus avellana* (hazel) which takes up to eight years to reach full productivity (19). Analysing and integrating these trees has required novel method development due to their genome size and complexity and is detailed later.

**Table 1.** Ensembl non-vertebrate growth/update 2019–2021

		Number of genomes				
Release	Date	Bacteria	Protists	Fungi	Plants	Metazoa
45	September 2019	44 048	237	1014	67	78
52	October 2021	31 332	237	1505	119	123
Change		–12 716	0	+491	+52	+45

Our metazoa resource has added sets of new or improved assemblies for pathogenic disease vectors including *Aedes aegypti* (vector for yellow fever, zika and chikungunya)*, Anopheles coluzzii* (vector for malaria), *Phlebotomus papatasi* (vector for leishmaniasis), six species of the *Glossina* complex (vector for sleeping sickness) (20) and the livestock pest *Stomoxys calcitrans* (21). Our twelve hosted Drosophila fly genomes have been refreshed to mirror those in FlyBase (22). Six strains of *Bemisia tabaci*, a cassava insect pest, are now available through our collaborative work with the African Cassava Whitefly Project (http://www.cassavawhitefly.org/) (23). Similarly, our collaboration with the Marine Invertebrate Models Database (MARIMBA) and CORBEL has brought two new marine metazoan genomes; *Actinia equina* (beadlet anemone) and *Clytia hemisphaerica* (a cnidarian). We also host a selection of well-studied nematode and flatworm genomes from the WormBase ParaSite project (https://parasite.wormbase.org) to enrich our comparative analysis. These include *Caenorhabditis elegans*; five other *Caenorhabditis*; parasites of humans and livestock including *Brugia malayi* (lymphatic filariasis) and *Loa loa* (African eye worm). Our fungal genomes coverage has increased significantly due to a new public archive import and 15 genomes originating from VEuPathDB’s fungal database, FungiDB, making Ensembl Fungi the most comprehensive collection of free/open access fungal genomes.

**Figure 1.**
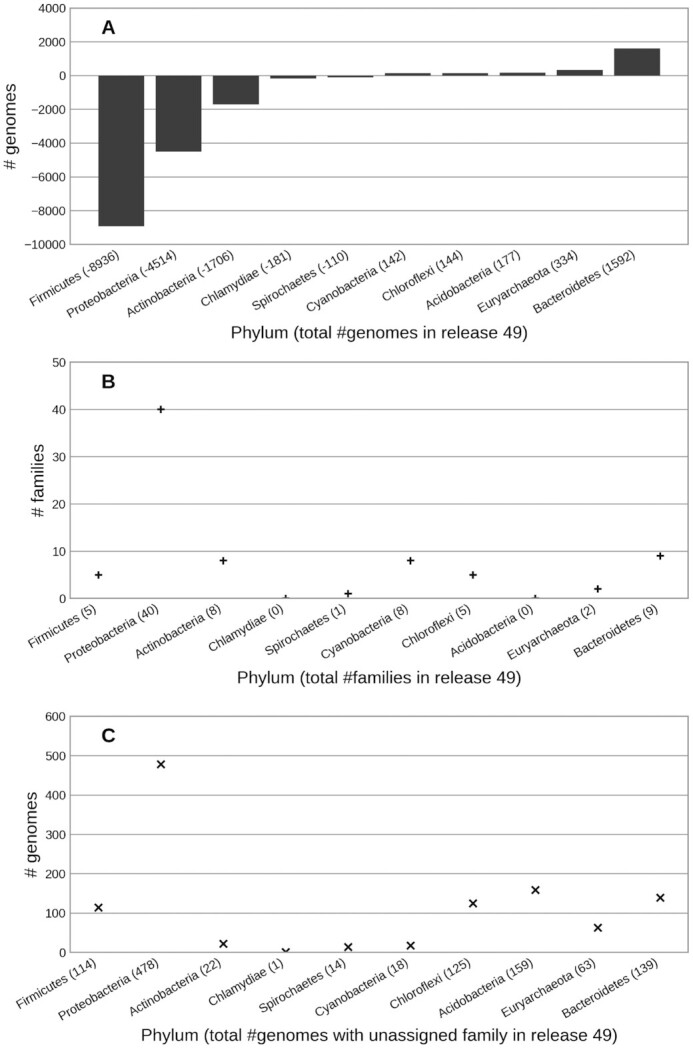
Shows the change in Ensembl Bacteria's collection, aggregated by our ten largest represented phylums, between releases 48 and 49. Component A shows the overall change in genome numbers in each phylum with over 15,000 genomes coming from three phylums. Component B demonstrates that overall family coverage within phylums has improved irrespective of the removal of genomes. Component C shows an increase in genomes without a known family with the majority occurring in Proteobacteria.

We chose to freeze our protists collection as we switched our focus towards identifying redundant genomes in our bacterial collection. We have adopted UniProt's prokaryotic proteome redundancy definitions, which removes closely related genomes based on the protein coding content (24). UniProt's methodology first creates a directed weighted graph of proteome similarity based on proteome content, taxonomic filtering and proteome size. It then finds the dominating set by repeatedly removing the weakest nodes until no more removals are possible. Adopting this approach has resulted in the removal of 12 716 genomes, whilst maintaining the coverage of 527 known bacterial families (Figure 1A and B). Cross referencing the removed genomes against NCBI’s family classification showed reductions in the *Streptococcaceae* (–5367), *Enterobacteriaceae* (–5278), *Staphylococcaceae* (–4877) and *Mycobacteriaceae* (–3547) families showing a previous over-representation in well studied bacterial families (Figure 1A). We also observed an increase of 957 genomes with no assigned taxonomy at the family level, raising the percentage of unclassified bacteria hosted within our resource to ∼10% (Figure 1C). All removed genomes remain accessible from our release 49 Ensembl Bacteria archive and FTP site. Further details can be found in our blog (https://www.ensembl.info/2020/09/21/ensembl-bacteria-updates/).

## GENOME ANNOTATION

The majority of genomes provided are annotated via a third-party data import from ENA records, large scale annotation providers including JGI (25), VEUPathDB, WormBase and FlyBase or directly from collaborators. We also conduct in-house annotation and make use of a parameter optimized version of Ensembl's automated gene annotation method. This was used to perform de-novo gene annotation of the aforementioned six *Bemisia tabaci* strains and recovered ∼90% of BUSCO Arthropoda/Insecta/Metazoa genes in five of the strains and ∼73% in the Uganda-1 strain showing suitability for use in non-vertebrate genomes. We also support community-based annotation projects using Apollo's web-based gene editing annotation tool and merge these new annotations back into our hosted gene sets (26).

We have updated our hosted variation annotation for six plants and 15 metazoans including *Triticum aestivum* (wheat), *Zea mays* (maize), *Culex quinquefasciatus* (southern house mosquito) and *Ixodes scapularis* (deer tick). All variation imports have their consequences pre-computed by Ensembl VEP. Our latest variation import also includes wheat linkage disequilibrium values and links to QTLs as found in CerealsDB (27). We also host variation directly from EVA, e.g. 43 million variants are available for *Phaseolus vulgaris* (common bean) (28). This provides a fast process to supplement genomes with variation and consequence predictions based on EVA submitted annotation.

## SCALING GENOME RESOURCES

In response to the recent increases in non-vertebrate genomes, we identified a need to accelerate researcher access to emerging data sets and scale our infrastructure to meet that demand. In 2020, Ensembl provided the ‘Ensembl Rapid Release’ website to support large-scale biodiversity studies and enables annotation release every two weeks, in contrast to its three-month integrated release cycle. *Clytia hemisphaerica* and *Actinia equina* were the first non-vertebrates to be made available via rapid release in 2020 and have been joined by *Vigna unguiculata* (black-eyed pea), *Cajanus cajan* (pigeon pea) and *Digitaria exilis* (fonio millet) representing crops of agricultural importance. We also redesigned our portal site (https://www.ensemblgenomes.org) to streamline user access to key genomes, switch our technology to the static site builder eleventy.js and to provide a new dynamic text search enabled by the EBI Search API (29).

## SUPPORTING PANGENOMES

Pangenome adoption is a growing area of interest and is considered a credible solution to reference biases and missing elements of a single reference genome. One such case is in *Triticum aestivum* where 12 150 genes were found to be missing from the reference assembly of the variety Chinese Spring Wheat, but were found in at least one of the 18 re-sequenced modern varieties (30, 31). To better model the wheat pangenome, we added nine new chromosome-scale wheat lines, alongside five additional scaffold-level assemblies published as part of the 10+ wheat genome consortium (http://www.10wheatgenomes.com/). Each assembly can be viewed individually via our genome browser or using our cultivar view, which reuses visualization views originally developed for mouse strains.

**Figure 2.**
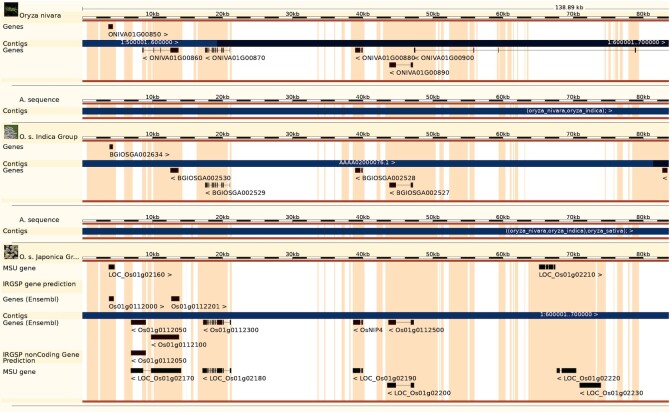
EPO multiple genome alignment visualization of chromosome 1 in three rice genomes: Oryza sativa indica Group (top), Oryza sativa japonica Group (middle) and Oryza glaberrima (bottom). Orange discontinuous blocks represent the areas of alignment across all three genomes. Each genome displays its genes and can be used to identify regions of uniqueness in each genome and identify potential areas of mis-assembly or mis-annotation. This alignment can be browsed at http://plants.ensembl.org/Oryza_nivara/Location/Compara_Alignments/Image?align = 9910;db = core;r = 1:586653–632276.

Generating high quality whole genome alignments is a key component in creating graph genomes. In preparation for increasing our pangenome support, we benchmarked Ensembl's existing whole genome aligner Enredo-Pecan-Ortheus (EPO) (32) against a set of 11 *Oryza* (rice) assemblies (Figure 2).

## LINKING GENOMES TO PREDICTED 3D STRUCTURE

**Figure 3.**
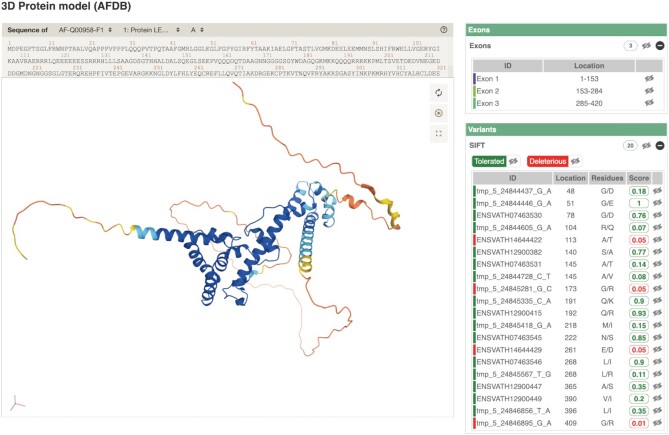
An AlphaFold 3D prediction for the Arabidopsis thaliana protein Q00958 (LFY: AT5G61850.1) displayed as a Richardson model using Mol*. The central panel annotates the model with regions of high confidence (blue) to low confidence (orange) with its protein sequence displayed above. The right hand panel enables highlighting of one or more exons, variants and protein features which are controlled by clicking on the eye icon. Variants can be turned on/off according to how deleterious or tolerated they are or individually. Only variants resulting in protein changes with SIFT scores are made available for display.

AlphaFold (33) has been a revolutionary advancement in 3D protein structure prediction and the release of AlphaFold DB in July 2021 (Varadi *et al.* in preparation) made available predictions across 17 non-vertebrate species providing previously unimaginable 3D proteome coverage. In the case of *Arabidopsis thaliana*, PDBe (34) contains 1661 experimental structures compared to 27 434 predicted structures available from AlphaFold DB. We used *A. thaliana* as a test for integration due to the availability of high-quality variant data and shared identity between ourselves and UniProt's reference proteome. We have successfully integrated AlphaFold models, visualized via Mol* (35), with exon and protein altering SIFT scored variants (Figure 3) (36). This view is available from our protein information page. We plan to expand our coverage to all available AlphaFold DB proteomes where possible across the non-vertebrate domain.

## PUBLIC ENGAGEMENT, OUTREACH AND TRAINING

We continue to offer training on our tools, interfaces, and APIs conducted virtually during the COVID-19 pandemic via video conferencing platforms. These platforms are used alongside participant interaction tools such as living documents (an open Google Doc where participants can type questions and answers), real-time messaging services, e.g. Slack and interactive polling software, e.g. Slido ensuring participants have multiple methods to communicate with trainers. There was also the return of the annual Wellcome Advanced course on fungal pathogen genomes co-developed with Wellcome, FungiDB, JGI and SGD (Saccharomyces Genome Database) and conducted virtually this year, after a break in 2020. A key teaching point of this course is the effective piecing together of features from multiple fungal resources to find the best answer to a biological question.

Finally, the global pandemic has also made public engagement increasingly difficult as social distancing requirements have challenged in-person interaction. Working in conjunction with the Cambridge University Botanic Garden, we co-developed ‘DNA in the Garden Trail’; a unique COVID-safe self-guided tour around the botanic gardens with a focus on plants hosted in Ensembl Plants (https://www.botanic.cam.ac.uk/education-learning/trails/dnatrail/). A companion application was built using Guidemap and offered a plant genomics quiz for families to complete as they used the trail.

## SOFTWARE ANALYSIS RESOURCES

Over the past two years, we have released two new non-vertebrate software resources; a collection of Ensembl Genomes data production workflows (https://github.com/Ensembl/ensembl-production-imported) and a set of analysis scripts for Ensembl Plant genomes (https://github.com/Ensembl/plant-scripts). These scripts are provided in a number of programming languages (Python, R, Perl) and detail common tasks using our programmatic interfaces and databases. We also released a *de novo* repeat analysis method for plant genomes, which uses a combination of repeat finders, repeat libraries, such as RepBase (37) or REdat (38), Red (39) and a curated set of transposable elements from a well characterized set of plants enabling fast and accurate annotation of new genomes (40). These new methods help to maintain sustainable genome analysis through accurate annotation of repetitive sequence.

## FUTURE PLANS

Whilst most of our annotation comes from third party imports, we have grown our ability to annotate a diverse range of non-vertebrate genomes in-house. Ensembl Rapid Release has been a vital component of this strategy enabling fast dissemination and is becoming our preferred method of distribution for newly annotated genomes. We plan to continue annotating non-vertebrate genomes in-house, expand data types available via Rapid Release and release a new scalable homology prediction method in collaboration with Ensembl. Genomes will still flow into our taxonomic sites based on their importance, scientific interest, broadening of our comparative analyses and when in-line with Ensembl's strategy for genome inclusion.

Continued growth in bacteria genomes necessitates a different strategy to handle duplication, inconsistencies of annotation and prioritize the needs of microbial researchers (41). As mentioned previously, 10% of bacteria lack a taxonomy, and this coupled with the continued growth in bacterial genomes derived from both isolate and environmental source will require further deployment of de-replication methods such as those used by Genome Taxonomy Database (42) to ensure our resources represent the breadth of bacterial diversity, yet continue to scale. Many newly submitted genomes lack gene annotation, and those that do have annotation can be of varying quality and/or show other issues e.g. inconsistent gene naming. To overcome these issues, we plan to re-annotate our hosted bacterial genomes and enrich them with functional annotations such as pathways and secondary metabolite gene clusters. We will also maintain existing annotations on key community reference genomes, e.g. *Escherichia coli K12* (U00096.3). Consistent high-quality annotation is key to enabling better downstream analysis such as developing new methods for deeper/broader functional annotation using machine learning. Our collaboration with MGnify (43)—EMBL-EBI’s metagenomics resource - will continue to expand. Briefly, we will focus on harmonizing the bacteria in Ensembl Genomes with the metagenome assembled genomes (MAGs) available in MGnify, through the adoption of common annotation pipelines, utilization of similar methods for the removal of genomic redundancy, i.e. GTDB (44, 45), and application of the same web presentation layers in both resources, making it easier for users to transition between the two resources. We will use the collections of Ensembl (isolate) genomes and MAGs as reference databases for determining their presence in metagenomes, to better understand the biological environments these genomes are found in. As the range of microbes presented in MGnify expands beyond prokaryotic microbes, we anticipate further synergies. Part of this effort will involve improving our coverage of protists in Ensembl Genomes.

Our efforts to merge Ensembl and Ensembl Genomes resources continues within the context of Ensembl's new infrastructure and website project (https://2020.ensembl.org). Of the seven genomes available through the new infrastructure, five are non-vertebrates; *Triticum aestivum, Caenorhabditis elegans, Saccharomyces cerevisiae, Plasmodium falciparum* and *Escherichia coli K12*. This puts non-vertebrate genomes at the centre of our future strategy, reflecting the increasing popularity of these genomes. Our efforts to reuse the rapid release infrastructure underlines that this strategy is not only possible but will create a better experience for researchers. We encourage those interested in shaping the future of this site to give feedback via our helpdesk and to sign up to our user experience sessions.

Finally, we expect significant progress in our support for pangenomes both in data processing and visualization. We plan to utilize our multiple sequence alignment methodology to construct genome graphs of rice and wheat cultivars increasing our support for pangenome analysis and visualization.
